# Genetic Fine Mapping and Genomic Annotation Defines Causal Mechanisms at A Novel Colorectal Cancer Susceptibility Locus in Han Chinese

**DOI:** 10.7150/jca.47189

**Published:** 2020-09-30

**Authors:** Kewei Jiang, Fengying Du, Liang lv, Hongqing Zhuo, Tao Xu, Lipan Peng, Yuezhi Chen, Leping Li, Jizhun Zhang

**Affiliations:** 1 Department of Gastrointestinal Surgery, Shandong Provincial Hospital, Cheeloo College of Medicine, Shandong University, Jinan, Shandong, China.; 2 Department of Gastrointestinal Surgery, Shandong Provincial Hospital Affiliated to Shandong First Medical University, Jinan, Shandong, China.; 3 Department of Gastroenterological Surgery, Peking University People's Hospital, Beijing, China.; 4 Department of General Surgery, Affiliated Hospital of Qingdao University, Qingdao, China.

**Keywords:** genome-wide association study, fine mapping, colorectal cancer, susceptibility loci, 5q23.3

## Abstract

Genome-wide association studies of colorectal cancer (CRC) have identified two risk SNPs. The characterization of these risk regions in diverse racial groups with different linkage disequilibrium structure would aid in localizing the causal variants. Herein, fine mapping of the established CRC loci was carried out in 1,508 cases and 1,482 controls obtained from the Han Chinese population. One distinct association signal was identified at these loci, where fine mapping implicated rs1010208 as a functional locus. Next, the candidate target genes of functional SNP rs1010208 were analyzed using data from TCGA databases by expression quantitative trait loci analysis method; the data from Peking University People's Hospital were utilized for verification. The dual-luciferase reporter system analysis confirmed that rs1010208 is a regulatory region that can be mutated to decrease the expression of *HINT1*, resulting in proliferation and invasiveness of CRC.

## Introduction

Colorectal cancer (CRC) is the third most common malignancy and the leading cause of cancer-related deaths worldwide, which accounts for > 1.8 million new cases and 861 thousand deaths every year 1. In the past few decades, an increasing trend of incidence and mortality has been observed in Asia including China 2. Thus, CRC has become a huge burden for society and individual families, and hence, elucidating the underlying pathogenesis and identifying new disease biomarkers is critical for the prevention and the early detection of CRC.

Genome-wide association studies (GWAS) does not indicate discovering of disease-causing genes or disease-causing mutations but rather locating the disease-causing mutations or genes; the true pathogenic mutations or genes are often identified in the vicinity of tag-single nucleotide polymorphisms (Tag SNPs) 3. GWAS loci are typically represented by a lead SNP with a robust signal of association in the region. However, lead SNPs might not directly impact the disease susceptibility but act as proxies for causal variants because of linkage disequilibrium (LD). Therefore, understanding the functional significance of these associated genetic loci is the biggest challenge post-GWAS 4.

Previously, our group identified two genetic loci related to the risk of CRC (rs12522693 at 5q23.3 and rs17836917 at 17q12) through the initial screening and validation by GWAS 5. The genes contained in these two susceptible regions play a major role in the formation and development of malignant tumors.

These two genetic loci were localized outside the DNA coding region and were not distinctly pathogenic. Therefore, further fine mapping of the above regions is essential, and the functional prediction of SNPs is utilized to identify the potential pathogenic or functional variations. The present study was conducted to explore the correlation between genetic variation and susceptibility to CRC in the Chinese Han population and to reveal the genetic susceptibility mechanism of CRC.

## Materials and Methods

### Ethics statement

All human-based studies were approved by the relevant institutional review boards and conducted according to the Declaration of Helsinki. All participants provided written informed consent.

### Studies and participants

Based on the previous three-stage GWAS project data, we established a library of validated DNA samples, complete sample information, and related operations 5. The validated DNA samples from 1,508 CRC cases and 1,482 controls, comprising two groups of independent samples, were used for targeted resequencing and genotyping. We set inclusion criteria as follow, all the CRC cases were histopathologically confirmed primary colorectal adenocarcinoma and they volunteered to participate in this project. Someone who had any prior history of other cancers, benign adenoma, familial history, chemotherapy, or radiotherapy was excluded. The controls were selected from individuals undergoing routine physical examinations in local hospitals or participating in a community-based screening program for non-infectious diseases conducted during the period when the cases were recruited. And individuals with a history of gastrointestinal malignancies were excluded. The controls were frequency-matched to the cases with respect to age (± 5 years) and gender. A volume of 5 mL venous blood was withdrawn from the individuals after the interview.

People who smoke more than one cigarette per day and smoke for more than one year in a row are considered smokers. Drinking twice a week and drinking more than 50 grams per drink, drinking more than 6 months of continuous drinking is considered a drinker. The participants were unrelated Han Chinese, and after training by the investigator, a questionnaire was used to conduct a one-to-one interview survey on the subjects. The collected data were entered using the two-track method, and the logistic check was corrected to perform statistical analysis.

### Imputation and association analysis

We selected a 200 kb region upstream and downstream of the two genetic loci (rs12522693 and rs17836917) found in the previous GWAS project 5 and 10 kb upstream of the candidate genes, *HINT1* and *CCL2*; then, the adjacent regions were combined to obtain two regions. According to the LD segment of the Chinese population from the HapMap database (HapMap Genome Browser release #27 Phase 1-, 2-, and 3-merged genotypes and frequencies), we extracted all the Tag SNPs from these two regions and filtered with R^2^ < 0.8 and minor allele frequency (MAF) in the Han Chinese (CHB) ≥ 0.05; the genotyping success rate was ≥ 0.90. (http://hapmap.ncbi.nlm.nih.gov/cgi-perl/gbrowse/hapmap27_B36/).

In order to identify the potential functional sites, we used the SNPinfo strategy to functionally annotate the screened sites and select the functional sites in the annotations 6, including non-synonymous SNPs, shear regulatory sites, stop codon, SNPs3D prediction, transcription factor binding site (TFBS) prediction, miRNA binding site prediction, and the genes in the vicinity. The online SNP SNAP software was used to analyze the linkage of the screened genetic loci.

### Genotyping and quality control

Genotyping of the selected SNPs was performed with a total of 1,979 samples using Affymetrix Axiom Genome-Wide CHB1 and CHB2 arrays that contained 1,280,786 SNPs chosen for the CRC-related projects. The Axiom array data underwent extensive quality control (QC) procedures carried out by Affymetrix as detailed in our previous study 5. Only SNPs that passed the QC were retained for subsequent analyses. After the application of these QC filters, the whole CRC dataset contained 1,898 subjects (932 cases and 966 controls) and 1,129,636 SNPs.

### Identification of distinct association signals in established GWAS loci

The SNPs identified by the targeted resequencing using DNA samples from 1,508 CRC cases and 1,482 controls were validated. Association analysis was performed using a logistic regression model with adjustment for age, sex, smoking, and drinking to calculate the correlation *P*-value, correlation odds ratio (OR), and 95% confidence interval (CI). Then, random-effect and fixed-effect meta-analysis were conducted to assess the pooled genetic effects in the validation stage.

### Prediction and verification of target genes for rs1010208

We obtained genomic annotation files for SNP genotype data and matched raw gene expression data of 146 patients with CRC assayed through The Cancer Genome Atlas (TCGA) database. An SNP with high LD (R^2^ > 0.9), risk SNP rs1010208, was selected as the proxy SNP, and the samples used for LD were from 1000 genome phase 1 data (CEU, CHB, JPT). Next, cis-expression quantitative trait loci (cis-eQTL) analysis was performed with rs1010208 using the transcripts within 1 Mb upstream and downstream, and the correlation of each pair of SNP-transcript expression was analyzed by a linear regression model with a *P*-value < 0.05 and false discovery rate (FDR) < 0.05.

Then, we obtained RNA-Seq data of 286 CRC tumors and 41 normal tissues from TCGA database for target-gene correlation studies. Based on the normalized data after chip analysis, a one-way ANOVA test was conducted to observe the differentially expressed genes in these two groups (*P*-value < 0.05).

Subsequently, real-time polymerase chain reaction (real-time PCR) was performed to detect the gene expression in 55 CRC patients undergone gastrointestinal surgery at the Peking University People's Hospital from May 2013 to August 2013 (characteristics of samples are listed in Supplementary Table S1).

### Dual-luciferase activity assay

We synthesized 965-bp nucleotide sequences containing the risk or protective allele of *HINT1* in both forward and reverse orientations. Then, complementary single-stranded oligos were annealed and subcloned into the minimal promoter-driven luciferase vector pGL3 using DH5α. The isolated clones were verified by sequencing and BLAST analysis. The cells were transfected using 1 μg pGL3 DNA vector harboring the protective or risk *HINT1* gene, or an equivalent amount of empty vector DNA with 100 ng SV40 DNA as a transfection control/well. After 48 h post-transfection, the cells were lysed and analyzed for Firefly and Renilla luciferase activities using the Dual-luciferase assay system according to the manufacturer's instructions in a half-volume 96-well plate on an Enspire Multimode Plate-Reader. Firefly luciferase activity (F) was normalized to the Renilla luciferase activity (R) for each well, and the results were expressed as a mean normalized activity relative to the empty vector-transfected cells.

### Functional verification of target gene *HINT1*

Supplementary Table S2 contains sample information for functional verification. We performed immunohistochemistry staining on these samples and analyzed the correlation between HINT1 protein expression and clinicopathological parameters in the CRC tissues by the chi-square test (Abcam: ab124912, 1:500). Kaplan-Meier method and Cox risk model were used for detecting the correlation between the disease and the primary clinical indicators.

SW480 and HCT116 cell lines were purchased from the Chinese Academy of Sciences Cell Bank (Shanghai, China) and cultured in RPMI1640 liquid medium (Gibico, USA) containing 10% fetal bovine serum (Gibico, USA). Human colonic fibroblast CCD-18Co (ATCC, CRL-1459) were set as a control and cultured in Dulbecoo minimum essential medium (DMEM) high-glucose medium (HyClone, SH30243.01B) supplemented with 10% fetal bovine serum (Gibco, USA). The cells were cultured in an incubator with 37 °C and 5% CO_2_, and other procedures were followed according to the general instructions. SW480, HCT116 and CCD-18Co cell lines were cultured and passaged at 90% confluence, after trypsinization at the logarithmic phase, the cells were sub-cultured in our standard medium until reaching 30% confluency. Transfected with LV-HINT1 overexpressing lentivirus, and western blot was used to verify the effect of overexpression (Abcam: ab124912, 1:1000). Subsequently, we performed a scratch test, an invasion test, an apoptosis test, and an MTT assay.

### Statistical methods

For stratified analysis, the heterogeneity test between the groups was based on the chi-square distribution Q test. The univariate analysis was performed prior to cis-eQTL analysis, and samples of normal distribution were subjected to paired T-test, or else rank-sum test was performed. A two-sided unpaired T-test was used to compare the luciferase activity between the alleles. The variables were compared by the T-test using the least-significant difference (LSD) method or one-way ANOVA test.

*P*-value < 0.05 and FDR < 0.05 were used as the criteria of statistical significance, and all statistical tests were two-sided. The statistical analyses were performed using Stata v11.0 and R software (version 3.5.1; The R Foundation for Statistical Computing).

## Results

### Characteristics of samples for fine mapping

The samples used in the validation stage included 1,482 controls and 1,508 cases from Beijing and Jiangsu regions. As shown in Table S3, no significant difference as detected in the age and gender between the case and control groups (*P*-value = 0.131 and 0.105, respectively). Also, and no significant difference was observed in the composition of smoking and drinking status between the control and cases group (*P*-value = 0.238 and 0.248, respectively).

### Genotyping results for validation sites

After the functional annotation of the screening loci by the SNPinfo strategy, we obtained 45 loci. SNPsnap online software was used for the linkage analysis. As a result, we obtained 10 functional sites, and the functional information is summarized in Supplementary Table S4. In the iPLEX MassARRAY genotyping map, the triangles on two sides indicate mixed genotypes, the squares in the middle are heterozygous, and the red dots are unclear. Figure S1 illustrates the genotyping of 10 SNPs in this study. The success rate of genotyping at 10 sites was > 98%.

### Correlation between verification loci and risk of CRC

As shown in Table 1, the genotype distribution of the control group at 10 sites was consistent with the Hardy-Weinberg equilibrium. SNP rs1010208 was significantly associated with the risk of CRC (OR = 1.15, 95% CI: 1.01-1.31, *P*-value = 0.029); however, no association was found between the other nine SNPs and the risk of CRC.

Logistic regression analysis revealed an increased risk of CRC (27%; adjusted OR = 1.27, 95% CI: 1.08-1.49) in individuals with heterozygous genotype (CT) as compared to the individuals carrying rs1010208 wild-type homozygous genotype (CC) after adjustment for gender, age, and smoking status. The log-added model showed that the allele T responded to the risk of CRC in a dose-dependent manner. For each additional T allele, the risk of CRC increased by an average of 14% (adjusted OR = 1.14, 95% CI: 1.00-1.30). Strikingly, the allele T is significant in the heterozygous type but not significant in the mutant homozygote, which might be attributed to the slight frequency in the homozygous mutation. However, no statistically significant association was observed between the other 9 loci and the risk of CRC (Supplementary Table 2).

### Stratified analysis of rs1010208 and the risk of CRC

We conducted a stratified analysis of rs1010208 based on age, gender, smoking status, and drinking status. As shown in Table 3, the effect of the rs1010208 CT genotype on increasing the risk of CRC was significantly associated with the drinking status (OR = 1.21, 95% CI: 1.05-1.40), and the drinking status increased the risk of CRC in individuals with heterozygous genotype (CT) by 21%. Other sites were not significantly associated with CRC at all stratifications.

### Acquisition of proxy SNPs and determination of target gene function

The association analysis conducted with the samples from the TCGA database retrieved 8 SNPs with a high degree of LD in rs1010208 (Supplementary Table S5).

**Figure 1:**
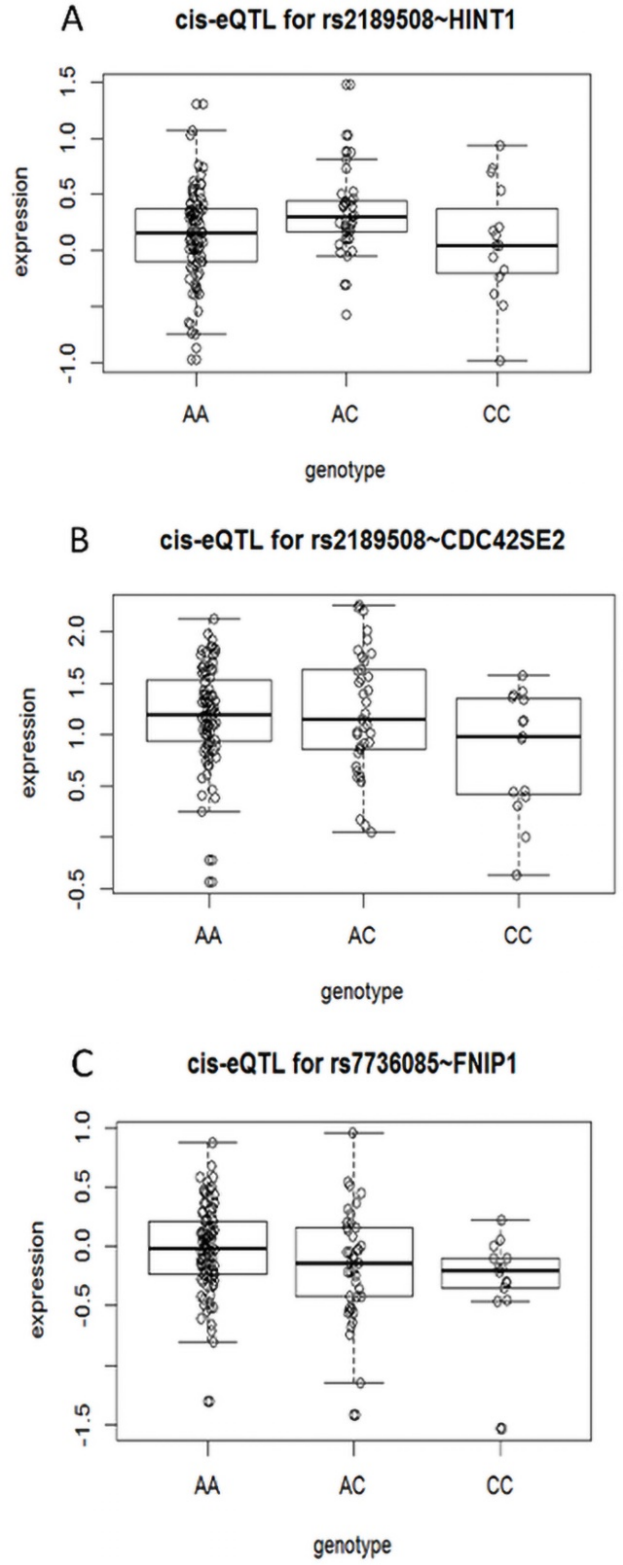
** Cis-eQTL correlation schematic.** (A) eQTL correlation between SNP rs2189508 and *HINT1*. (B) eQTL correlation between SNP rs2189508 and *CDC42SE2*. (C) eQTL correlation between SNP rs7736085 and *FNIP1*.

The eQTL analysis of proxy SNP revealed that 3 genes, *HINT1*, *CDC42SE2*, and *FNIP1*, are statistically different, respectively; especially 4 proxy SNPs were associated with *CDC42SE2* in cis-eQTL (Supplementary Table S6 and Figure 1). However, we proposed that the data used in this analysis are collected from the European and American populations and need further verification in the Chinese population.

Based on the normalized TCGA RNA-Seq data, we found that among the candidate target genes, the expression of *HINT1*, *CDC42SE2* and *FNIP1* differed between the cancer tissues and the normal tissues (*P*-value = 0.0172, *P*-value <0.0001 and *P*-value <0.0001, respectively, shown in Supplementary Fig S2), however, in the TCGA data, tumor and normal tissues data are not paired.

Next, cis-eQTL correlation analysis was performed with 55 pairs of samples from Peking University People's Hospital. The results showed a cis-eQTL correlation between the SNP rs1010208 genotype and the expression of *HINT1*, thereby, indicating that the target gene of SNP rs1010208 is *HINT1*. Nonetheless, no cis-eQTL correlation was detected between the SNP rs1010208 genotype and the expression of *CDC42SE2*. Thus, we observed that the expression of *HINT1* and *CDC42SE2* mRNA in cancer tissues was lower than that in the adjacent tissues (*P*-value < 0.001, Supplementary Figure S3). Subsequently, we validated the target gene of SNP rs1010208 in the specimens from Peking University People's Hospital and found that *HINT1* not only presented a cis-eQTL correlation with SNPrs1010208 but also a statistically significant difference in the expression between tumor and adjacent tissues.

### Role of SNP rs1010208 in target genes

We spliced the SNP rs1010208, the promoter region, and the fluorescent reporter group in a specific order referring to the sequence in Supplementary Figure S4 for four groups. Thus, it can be concluded from the data in Supplementary Figure S5 that group 1 had low F/R values because it consisted of only the fluorescent reporter group. However, the reason for higher F/R values from group 2 than those from group 3 or 4 requires an in-depth investigation. The F/R values from groups 3 and 4 were statistically different between the groups in both HCT116 and SW480 cells (*P*-value < 0.01). The results of both cell lines were consistent, indicating a difference in the expression of the target gene *HINT1* between the wild-type and mutant in the regulatory region. In summary, the mutations in the regulatory region, SNP rs1010208 downregulates the expression of the target gene *HINT1*.

### Function of the target gene *HINT1*

Next, we performed immunohistochemistry staining on 110 samples from Peking University People's Hospital to evaluate the expression of *HINT1* in tumor tissues. *HINT1* was expressed in both cytoplasm and nucleus, and significant differences were detected in the expression from patients with different stages of the tumor (*P*-value = 0.011; characteristics of the samples are listed in Supplementary Table S2, and the expression of *HINT1* in tissues is shown in Supplementary Figure S6). Kaplan-Meier survival analysis showed a significantly high survival rate of patients with high *HINT1* expression than that of those with low *HINT1* expression (*P*-value = 0.013; Supplementary Figure S7). Additionally, Cox multivariate prognostic analysis showed that the level of *HINT1* expression and lymph node metastasis status were independent factors for the CRC patient in prognosis (*P*-value < 0.01). The expression of *HINT1* in patients with different stages of tumor varies and is related to the prognosis, rendering it to be a potential target for drug treatment.

**Figure 2:**
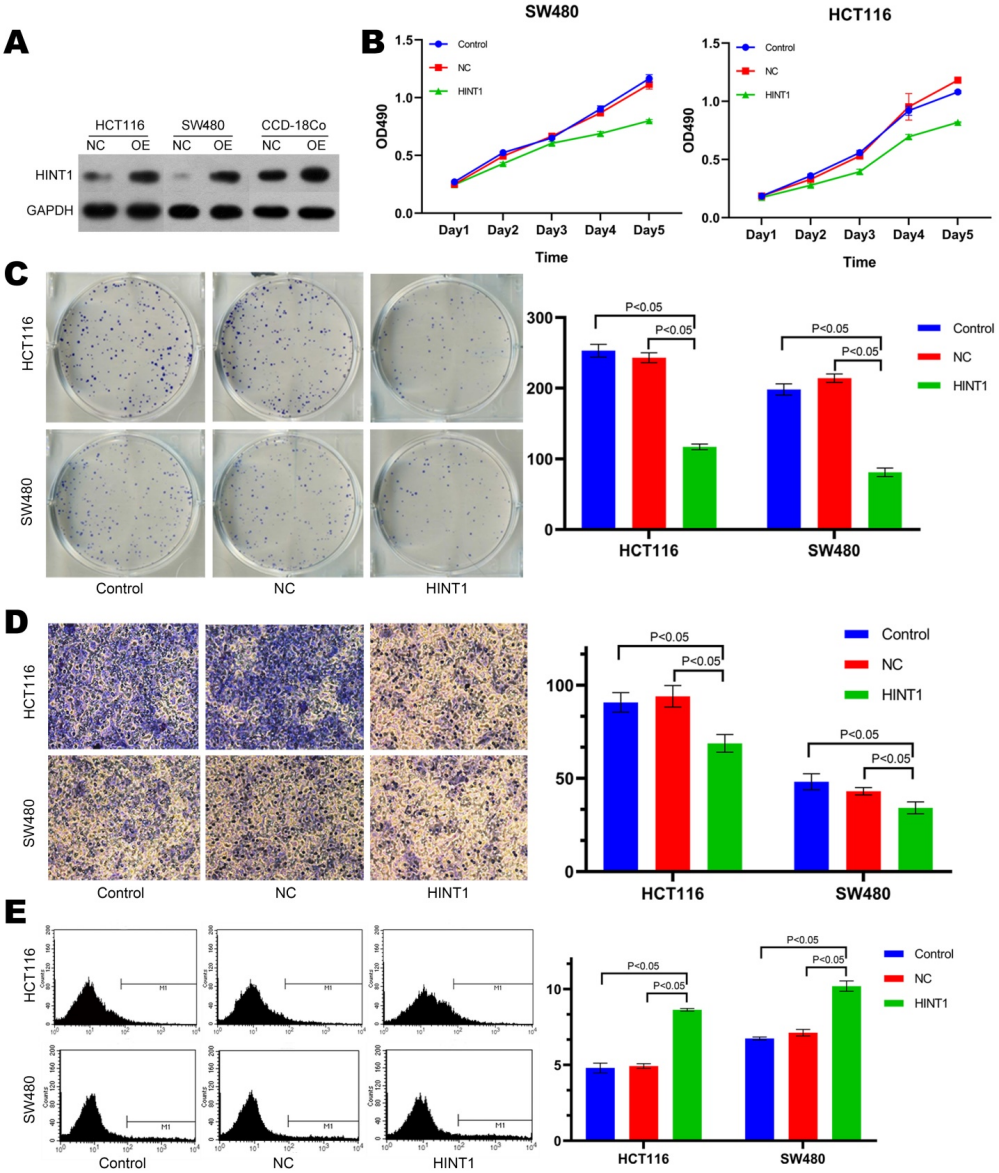
** Effect of *HINT1* on apoptosis in HCT116 cell line and SW480 cell line.** (A) Changes in expression level of *HINT1* in HCT116, SW480 and CCD-18Co cells after transfection. (B) Effect of *HINT1* gene on the proliferation of HCT116 and SW480 cells. (C) Effect of *HINT1* gene on the formation of HCT116 and SW480 cells. (D) Effect of *HINT1* gene on the invasion of HCT116 and SW480 cells. (E) Effect of *HINT1* gene on the apoptosis of HCT116 and SW480 cells.

HCT116, SW480 and CCD-18Co cell lines were transfected with overexpressing lentivirus harboring the fluorescent *HINT1* for 48 h. After digesting the cells, total protein was extracted and subjected to Western blotting with negative transfection as a control. The expression level of *HINT1* in three cell lines was significantly different from that in the control group (NC) as shown in Figure 2A (*P*-value < 0.05). Subsequently, the MTT assay demonstrated that there was no difference in cell proliferation after the transfection of *HINT1*-overexpressing lentivirus compared with at 0 h. After 24, 48, 72, and 96 h, the overexpression of *HINT1* significantly inhibited the proliferation of HCT116 and SW480 cell lines (*P*-value < 0.05, Figure 2B), the same result was also observed in the MTT assay of CCD-18Co cells (*P*-value < 0.05, Supplementary Figure S8A). In clone formation assay, after overexpression of *HINT1*, HCT116, SW480 and CCD-18Co cell lines all showed significant inhibitory effects (*P*-value < 0.05, Figure 2C and Supplementary Figure S8B). Next, we inoculated the cell lines and performed Transwell assay and Annexin V-APC single staining method to detect the ability of invasion, and apoptosis of the cell lines. Furthermore, the overexpression of *HINT1* inhibited the invasion ability and increased the apoptosis of HCT116 and SW480 cell lines as compared to the control group (*P*-value < 0.05, Figure 2D and 2E). In the assay of CCD-18Co, it was found that cells could not pass through the membrane of transwell chamber, regardless of whether *HINT1* overexpressed. However, after overexpression of *HINT1*, the apoptotic rate of CCD-18Co also increased (*P*-value < 0.05, Supplementary Figure S8C).

## Discussion

Screening for specific genetic variations that can truly explain the disease is challenging. We can integrate GWAS data with eQTL data to help us identify causal-related SNPs 7, 8. In this study, we performed eQTL analysis using data from the TCGA database and validated potential functional SNPs in 30 matched cases from the GEO database and 55 cases from Peking University People's Hospital. We finally demonstrated that rs1010208 in the gene *HINT1* was significantly associated with CRC risk in the Chinese population. The results of this study suggest that rs1010208 may reduce* HINT1* expression by affecting TF binding upstream of the gene, resulting in a lower risk of CRC. The dual-luciferase reporter assay confirmed the potential function of rs1010208, and the SNP rs101028 mutation in the regulatory region down-regulated the expression of the target gene *HINT1*.

*HINT1* belongs to the evolutionarily conserved HIT protein family and contains at least three members: *HINT1*, *FHIT* and *GALT* 9. Previous studies have shown that *HINT1* is a haploid-insufficient tumor suppressor gene 10, 11. *HINT1* interacts with Pontin and Reptin to inhibit T cell-mediated *WNT* transcription 12, 13. *HINT1* also up-regulates the expression of p53 and Bax (pro-apoptotic factor) and down-regulates the expression of Bcl-2 (apoptosis inhibitor), thereby participating in the cell apoptosis process 13. In some cancers, the transcript of *HINT1* is silenced or down-regulated, which confirms its important role in tumor formation.* HINT1* inhibits the Wnt/β-catenin pathway in colorectal cancer cells and is associated with microphthalmia-related transcription factor activity in mast cells 14-18. Genovese et al. found that *HINT1* inhibits the transcriptional activities of *MITF* and β-catenin, both of which are immunoprecipitated with anti-HINT1-specific antibodies in malignant melanoma cells 18, 19. The role of *HINT1* in melanoma cells may be achieved by promoting the formation of non-functional complexes with oncogene transcription factors such as *MITF* and β-catenin 18, 19. In addition to its inhibitory effect on tumor formation, *HINT1* also plays a role in many pathophysiological states 20-23.

However, we confirmed that *HINT1* is a tumor suppressor gene in colon cancer cells and there are still some limitations in our study. The area comprised in this study for fine mapping studies is limited, and no sites for remote regulation and rare mutations have been fine mapped in the current study, especially in intergenic and genetic desert areas 24. Therefore, several functional sites or pathogenic sites are not yet detected, and thus, it is necessary to perform deep resequencing of these two regions to identify additional pathogenic sites. On the other hand, functional sites or pathogenic sites are related to the genetic difference between the population from which the SNPinfo database is derived and the Han population. These phenomena need further investigation of the genetic susceptibility mechanism underlying CRC.

After clarifying the target gene and its function, the next step is to explore the molecular mechanism of CRC. The disease-related SNP values included in NHGRI showed a moderate risk effect, and the correlation between complete knockout and heterozygous knockout needs to be considered when using disease model validation 25. ZFN, TALEN, and CRISPR/Cas are the best ways to solve the above problems 26. Thanks to advances in programming techniques, researchers are able to achieve precise modifications at the nucleotide level to clarify the functional significance of SNP mutations.

## Conclusion

Fine mapping studies were performed on the susceptible regions, 5q23.3 and 17q12, which harbored the two susceptible loci rs12522693 and rs17836917 associated with the risk of CRC in the GWAS mentioned above in the Han population. Consequently, a novel potential functional locus, rs1010208 (chromosome 5), associated with susceptibility to CRC, downregulates the expression of the target gene *HINT1*, thereby inducing the proliferation and invasion of CRC. Together, the results of the present study provide a guiding significance for the genetic susceptibility mechanism underlying the CRC.

## Supplementary Material

Supplementary figures and tables.

## Figures and Tables

**Table 1:** Results of association analysis of verification loci

Locus	Base change^a^	HW^b^	MAF^c^ control/case	OR (95% CI)^d^	*P*-value
rs10068403	G/A	0.483	0.720/0.729	1.07 (0.96-1.20)	0.214
rs1029749	C/A	0.937	0.454/0.547	1.01 (0.91-1.11)	0.927
**rs1010208**	**C/T**	**0.362**	**0.577/0.815**	**1.15 (1.01-1.31)**	0.029^*^
rs33919	G/C	0.967	0.792/0.567	0.96 (0.87-1.07)	0.465
rs1023527	T/A	0.902	0.550/0.534	0.94 (0.85-1.04)	0.206
rs11658161	A/G	0.579	0.939/0.941	1.04 (0.84-1.29)	0.728
rs16969118	T/C	0.106	0.901/0.901	0.96 (0.81-1.14)	0.653
rs17837023	G/A	0.220	0.559/0.553	0.98 (0.88-1.08)	0.671
rs2189333	G/C	0.451	0.674/0.680	1.03 (0.92-1.15)	0.600
rs28935	T/C	0.933	0.550/0.560	1.04 (0.94-1.15)	0.442

^a^ Base change: High frequency/low-frequency allele; ^b^ Hardy-Weinberg equilibrium analysis in the control group; ^c^ MAF: Small allele frequency (case/control); ^d^ OR of the cumulative ratio logistic regression analysis, 95% CI.

**Table 2:** Correlation between verification loci and risk of CRC

Loci	Case group		Control group		OR (95% CI)^a^	*P*-value^a^
	N	%	N	%		
**rs10068403**	1509		1482			
GG	767	50.8	789	53.2	1.00	
AG	604	40.0	573	38.7	1.11 (0.95-1.30)	0.198
AA	126	8.3	114	7.7	1.11 (0.84-1.48)	0.457
A ^†^					1.08 (0.96-1.21)	0.197
**rs1029749**	1509		1482			
CC	441	29.2	438	29.6	1.00	
CA	736	48.8	728	49.1	1.02 (0.86-1.21)	0.847
AA	305	20.2	300	20.2	0.96 (0.79-1.22)	0.893
A**^†^**					1.02 (0.92-1.13)	.0716
**rs1010208**	1509		1482			
CC	937	62.1	984	66.4	1.00	
CT	502	33.3	435	29.4	1.27 (1.08-1.49)	**0.004**
TT	61	4.0	56	3.8	1.02 (0.70-1.52)	0.885
T**^†^**					1.14 (1.00-1.30)	**0.048**
**rs33919**	1509		1482			
GG	502	33.3	474	32.0	1.00	
CG	721	47.8	725	48.9	0.93 (0.79-1.10)	0.415
CC	273	18.1	276	18.6	0.93 (0.75-1.16)	0.538
C**^†^**					0.98 (0.88-1.08)	0.644
**rs1023527**	1509		1482			
TT	465	30.8	418	28.2	1.00	
AT	717	47.5	735	49.6	0.86 (0.73-1.03)	0.100
AA	315	20.9	319	21.5	0.87 (0.70-1.07)	0.189
A**^†^**					0.92 (0.83-1.02)	0.128
**rs11658161**	1509		1482			
AA	1319	87.4	1305	88.1	1.00	
AG	174	11.5	167	11.3	1.05 (0.83-1.32)	0.665
GG	5	0.3	4	0.3	1.15 (0.29-4.65)	0.835
G**^†^**					1.05 (0.84-1.30)	0.680
**rs16969118**	1509		1482			
TT	1232	81.6	1190	80.3	1.00	
CT	250	16.6	275	18.6	0.91 (0.75-1.11)	0.347
CC	19	1.3	9	0.6	2.12 (0.99-4.52)	0.052
C**^†^**					0.97 (0.81-1.15)	0.711
**rs17837023**	1509		1482			
GG	473	31.3	461	31.1	1.00	
AG	731	48.4	703	47.4	0.98 (0.83-1.16)	0.833
AA	297	19.7	305	20.6	0.91 (0.73-1.12)	0.358
A**^†^**					0.98 (0.89-1.09)	0.733
**rs2189333**	1509		1482			
GG	674	44.7	687	46.4	1.00	
CG	654	43.3	628	42.4	1.09 (0.93-1.28)	0.278
CC	158	10.5	157	10.6	1.02 (0.79-1.32)	0.858
C^†^					1.04 (0.94-1.16)	0.468
**rs28935**	1509		1482			
TT	443	29.4	461	31.1	1.00	
CT	763	50.6	723	48.8	1.10 (0.92-1.30)	0.289
CC	286	19.0	286	19.3	1.10 (0.89-1.37)	0.386
C**^†^**					1.04 (0.94-1.15)	0.464

^a^ Corrected by age, gender, and smoking and drinking status; **^†^**The log-added model, only the genotype enters the model as a determinant variable.

**Table 3:** Stratified analysis of rs1010208 and risk of CRC

Factors	rs1010208 (T > C)^a^	
	OR (95% CI)	*P*-value
**Age (years)**		
< 60	1.18 (0.99-1.40)	0.060
≥ 60	1.14 (0.94-1.39)	0.196
**Gender**		
Male	1.15 (0.97-1.36)	0.110
Female	1.18 (0.96-1.47)	0.112
**Smoke**		
Never	1.15 (0.99-1.34)	0.055
Yes	1.08 (0.82-1.42)	0.575
**Drink**		
Never	0.94 (0.67-1.31)	0.708
Yes	1.21 (1.05-1.40)	0.011^*^

^a^Age and gender adjusted in the logistic regression model.

## Abbreviations

CRC: Colorectal cancer
GWAS: Genome-wide association studies
Tag SNPs: Tag-single nucleotide polymorphisms
LD: Linkage disequilibrium
MAF: Minor allele frequency
TFBS: Transcription factor binding site
QC: Quality control
OR: Odds ratio
CI: Confidence interval
TCGA: The Cancer Genome Atlas
cis-eQTL: cis-expression quantitative trait loci
FDR: False discovery rate
real-time PCR: Real-time polymerase chain reaction
LSD: Least-significant difference
