# Supplementary material for: Genetic Fine Mapping and Genomic Annotation Defines Causal Mechanisms at A Novel Colorectal Cancer Susceptibility Locus in Han Chinese
Source: J Cancer. 2020 Sep 30;11(23):6841–9. doi: 10.7150/jca.47189 (PMC7592009; doi:10.7150/jca.47189)
Supplement: Supplementary file 1 — Supplementary figures and tables. [file jcav11p6841s1.pdf]

Supplementary Material

Supplementary Figures

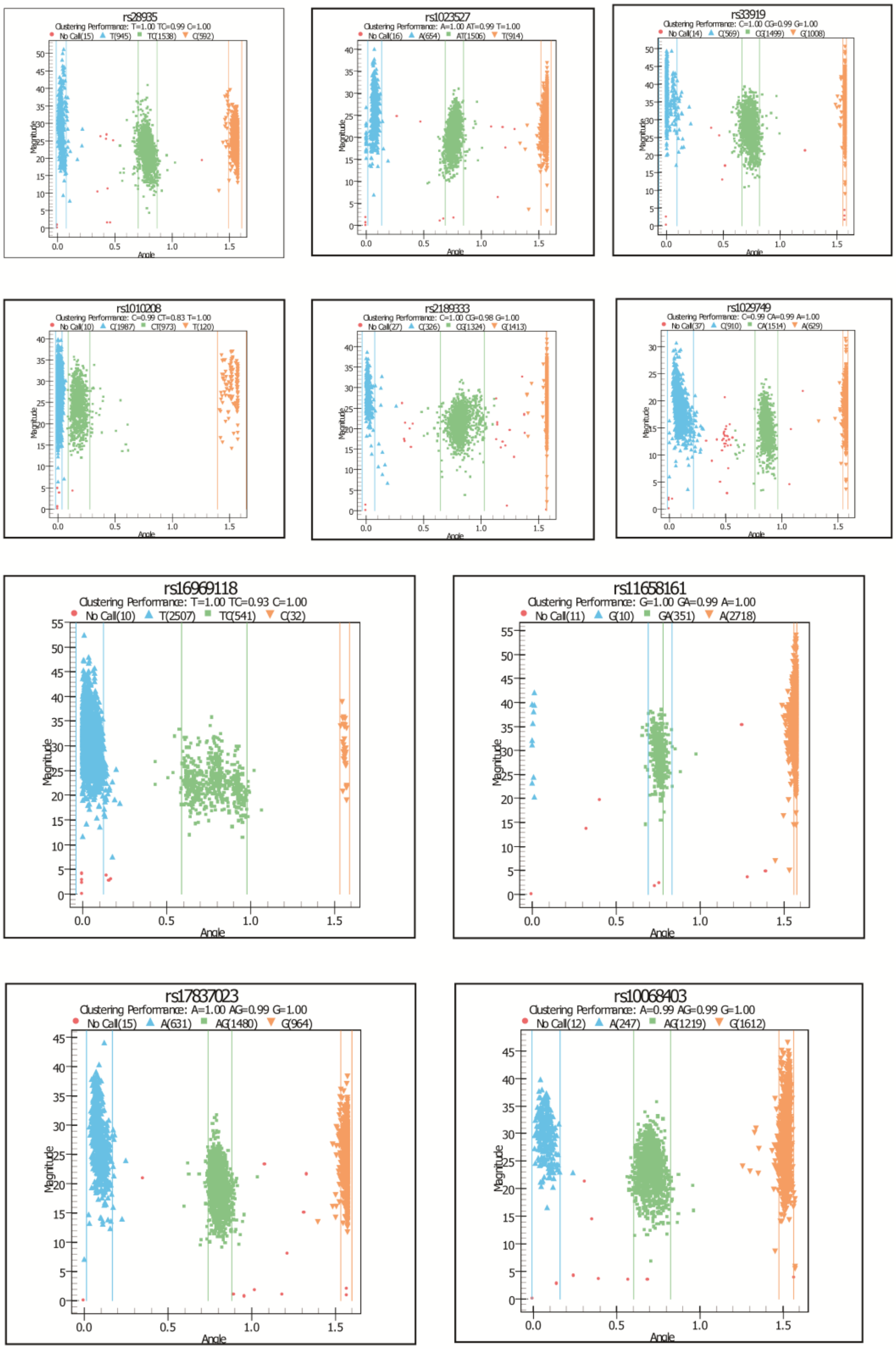

Supplementary Figure S1. Genotyping of Sequenom MassARRAY for SNPs.

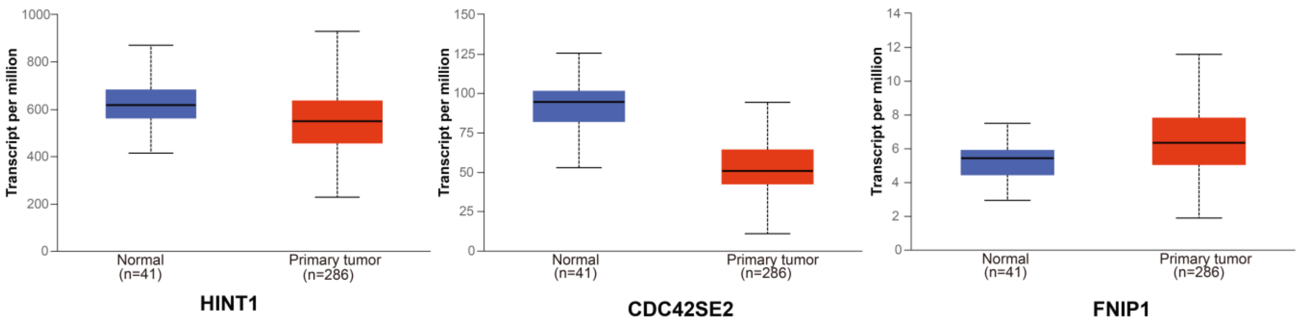

Supplementary Figure S2. mRNA expression in TCGA database.

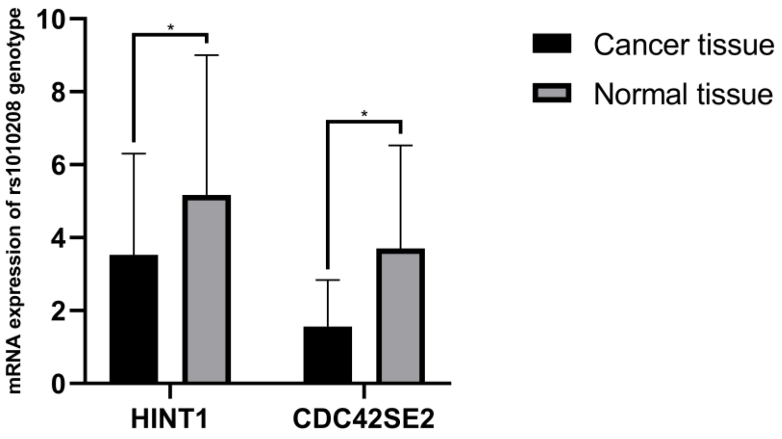

Supplementary Figure S3. the difference between *HINT1* and *CDC42SE2* expression.

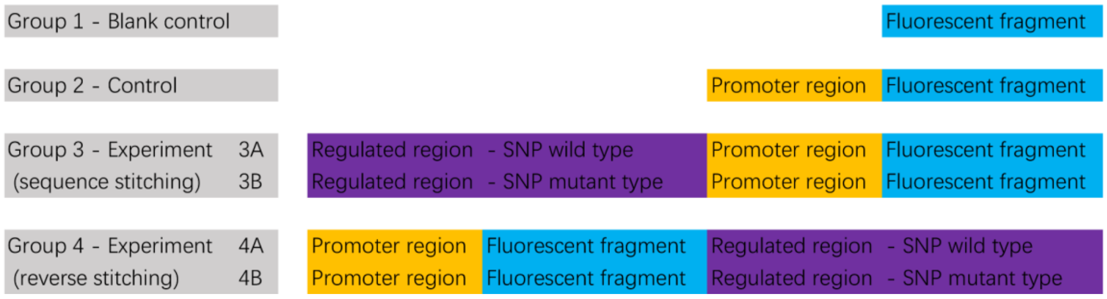

Supplementary Figure S4. Splicing order and dual-luciferase assay grouping.

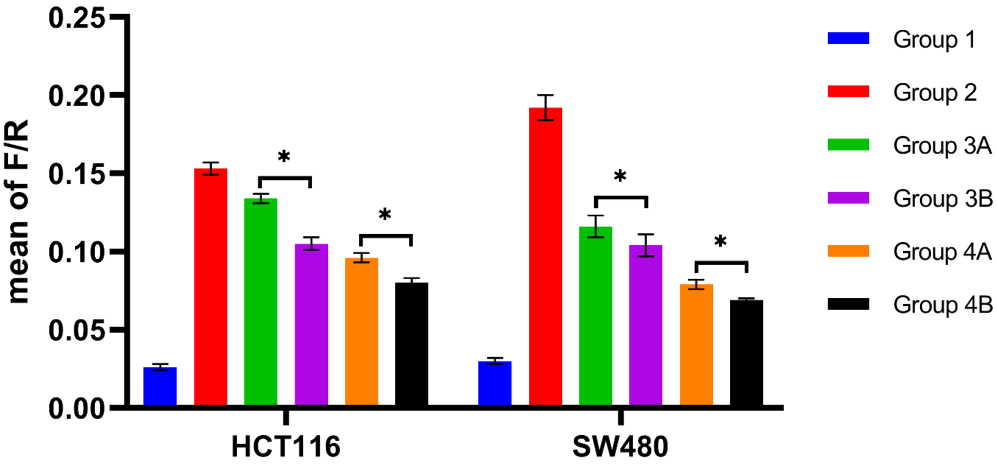

Supplementary Figure S5. Result of dual-luciferase assay.

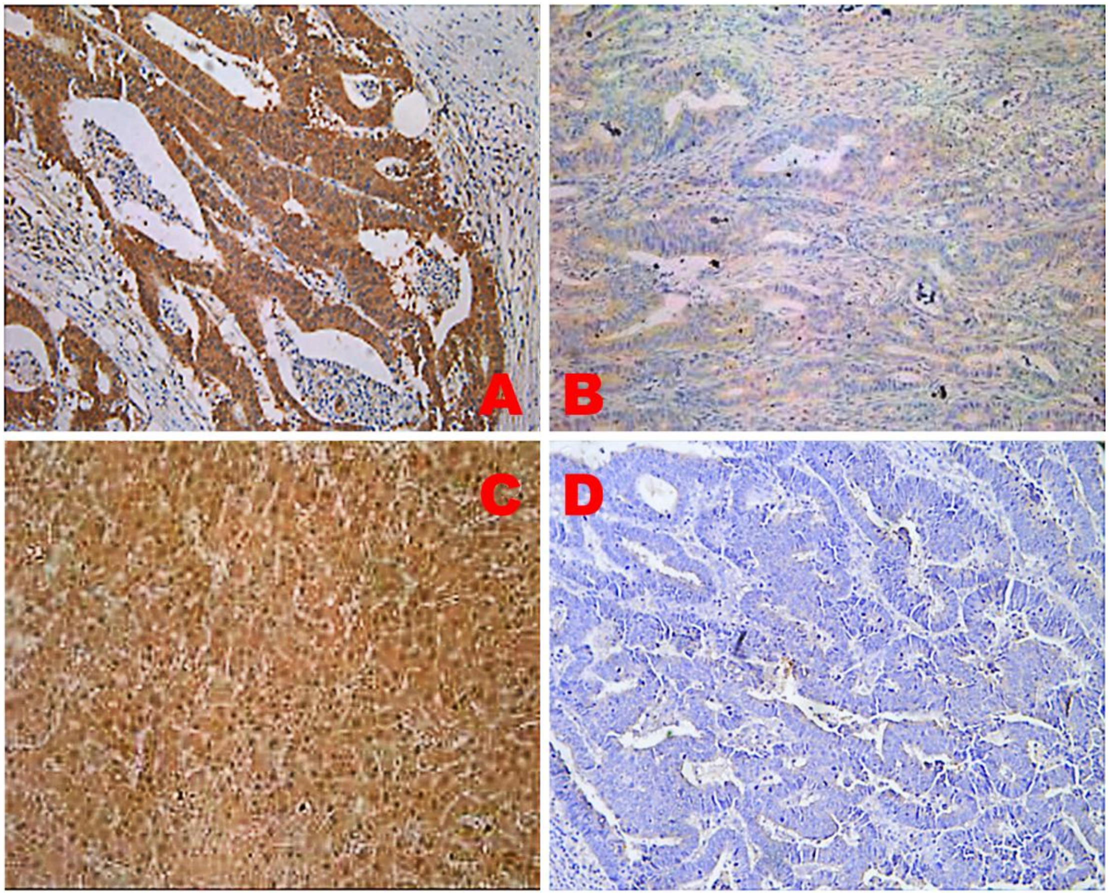

Supplementary Figure S6. Expression of *HINT1* in tissues. (A) High expression of HINT1 in tissues (×100). (B) Low expression of HINT1in tissues (×100). (C) HINT1 positive control in cervical cancer tissues (×100). (D) PBS as negative control instead of primary antibody (×100).

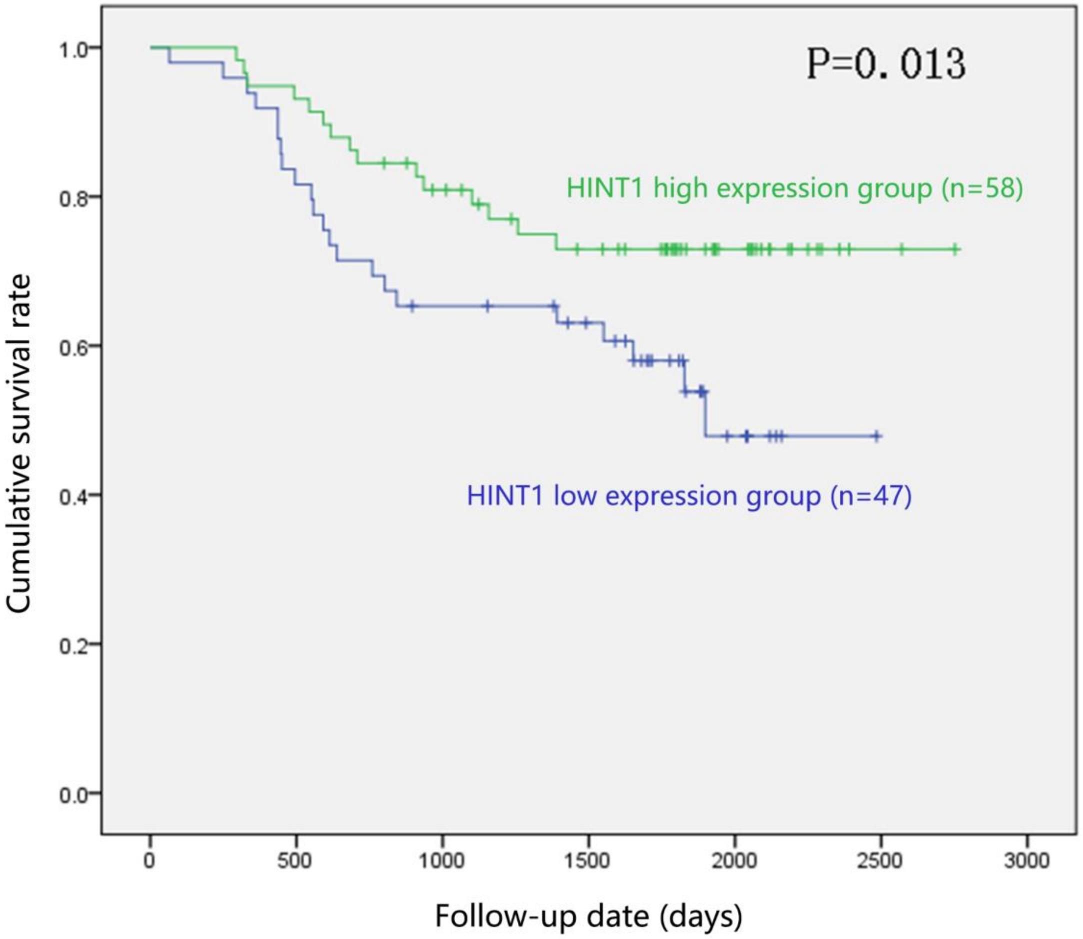

Supplementary Figure S7. Association between *HINT1* and patient survival.

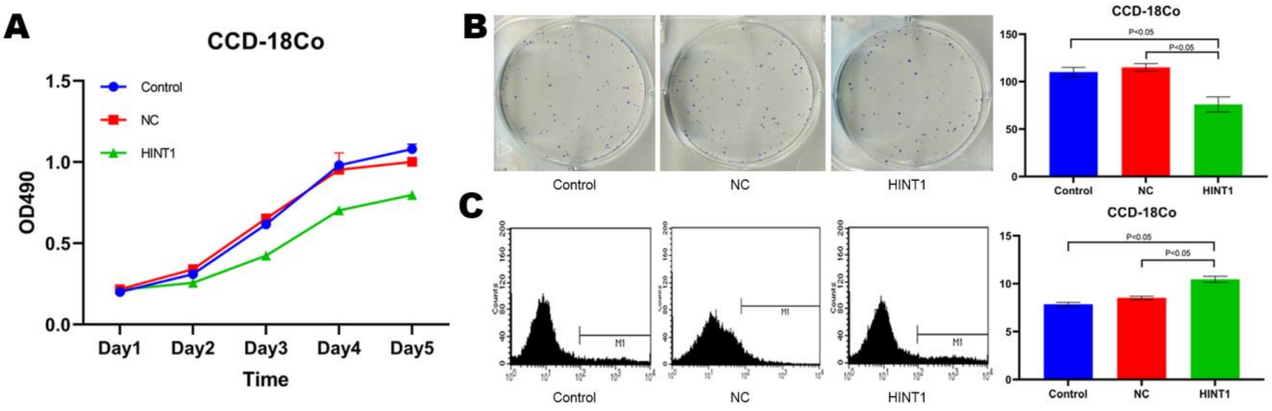

Supplementary Figure S8. *In vitro* experiment after CCD-18Co overexpression of *HINT1*.

Supplementary Tables

Supplementary Table S1. Clinical characteristics of 55 patients

Characteristics		Case number	Ratio (%)
Gender	Male	37	67.3%
	female	18	32.7%
Age	< 60	19	34.5%
	≥ 60	36	65.5%
Maximum tumor diameter	< 5 cm	28	50.9%
	≥ 5 cm	27	49.1%
Differentiation	high	4	7.3%
	medium	39	70.9%
	low	12	21.8%
T stage	T1	3	5.5%
	T2	10	18.2%
	T3	29	52.7%
	T4	13	23.6%
N stage	N0	27	49.1%
	N1	14	25.5%
	N2	14	25.5%
TNM stage	I	9	16.4%
	II	18	32.7%
	III	25	45.5%
	IV	3	5.5%

Supplementary Table S2. Clinical characteristics of 110 patients

Factors	Cases	Expression of *HINT1*		*P*-value
		Low (n = 49)	High (n = 58)	
Age				0.084
>60	74	38	36	
≤60	33	11	22	
Gender				0.057
Male	66	35	31	
Female	41	14	27	
Degree of differentiation				0.819[a]
High	25	8	17	
Medium	68	34	32	
Low	14	3	9	
Maximum diameter of tumor				0.737
> 5 cm	44	21	23	
≤ 5 cm	63	28	35	
Depth of tumor invasion				0.942
T1-T2	15	7	8	
T3-T4	92	42	50	
Lymph node metastasis				0.117
N0	59	23	36	
N+	48	26	22	
TNM staging				**0.050**
I + II	59	22	37	
III + IV	48	27	21	

[a] χ^2 test with Linear-by-Linear Association

Table S3. Characteristics of samples

Factors	Control group (= 1482) N (%)	Case group (= 1508) N (%)	χ^2	*P*-value [a]
Age (years)			2.285	0.131
≤ 60	869 (58.6)	843 (55.90)		
> 60	613 (41.4)	665 (44.10)		
Gender			2.627	0.105
Male	865 (58.37)	924 (61.27)		
Female	617 (41.63)	584 (38.73)		
Smoke			1.395	0.238
Never	1145 (77.26)	1192 (79.05)		
Yes	337 (22.74)	316 (20.95)		
Drink			1.337	0.248
Never	1234 (83.27)	1279 (84.81)		
Yes	248 (16.73)	229 (15.19)		

[a] two-sided χ^2 test

Supplementary Table S4. Functional information of the substantiated verification sites

Site	Chromosome	Location	Allele	TFBS	miRNA (miRanda)	miRNA (Sanger)	nsSNP	Nearby Gene
rs10068403	5	129548578	A/G	--	--	--	**Y**	*CDC42SE2*, *HINT1*
rs1029749	5	129892947	A/C	**Y**	--	--	--	*CHSY3*, *HINT1*
rs1010208	5	130353838	C/T	**Y**	--	--	--	*CDC42SE2*, *HINT1*
rs33919	5	129267239	C/G	**Y**	--	--	--	*CHSY3*, *HINT1*
rs1023527	17	29405724	A/T	**Y**	--	--	--	*ACCN1*, *CCL*
rs11658161	17	29426263	A/G	**Y**	--	--	--	*ACCN1*, *CCL*
rs16969118	17	29401787	C/T	**Y**	--	--	--	*ACCN1*, *CCL*
rs17837023	17	29423118	A/G	**Y**	--	--	--	*ACCN1*, *CCL*
rs2189333	17	29425147	C/G	**Y**	--	--	--	*ACCN1*, *CCL*
rs28935	17	28364557	C/T	--	**Y**	**Y**	--	*ACCN1*, *CCL*

Supplementary Table S5. SNPs with a high degree of LD in rs1010208

SNPs	Allele A	Allele B	CEU (r^2)	CHB (r^2)	JPT (r^2)	Distance
rs7736085	A	C	1	1	1	1414
rs6595983	A	G	1	1	1	9816
rs6595989	C	T	1	1	1	33276
rs973900	G	T	1	1	1	36528
rs12719432	C	T	1	1	1	28356
rs10478956	C	G	1	1	0.96	7425
rs6595984	A	G	1	1	1	9945
rs2189508	A	C	1	1	0.92	21730

Supplementary Table S6. Results of cis-eQTL analysis

SNP	Gene	*P*-value	FDR
rs2189508	*HINT1*、*CDC42SE2*	0.0017	0.0311
rs10040560	*FNIP1*	0.0013	0.0311
rs12719432	*CDC42SE2*	0.0022	0.0311
rs6595989	*CDC42SE2*	0.0028	0.0337
rs6883635	*CDC42SE2*	0.0006	0.0311
